# Examining the Longitudinal Associations of Social Support in relation to Cognitive Performance and ADRD Blood‐Based Biomarkers in the HABS‐HD Cohort

**DOI:** 10.1002/alz70861_108355

**Published:** 2025-12-23

**Authors:** Jillian K. Lee, Leigh A. Johnson, James Hall, Trey R. Bateman, Sid E. O'Bryant, Michelle M Mielke

**Affiliations:** ^1^ Wake Forest University School of Medicine, Winston‐Salem, NC USA; ^2^ Institute for Translational Research, University of North Texas Health Science Center, Fort Worth, TX USA; ^3^ University of North Texas Health Science Center, Fort Worth, TX USA; ^4^ Division of Public Health Sciences, Wake Forest University, School of Medicine, Winston‐Salem, NC USA

## Abstract

**Background:**

There is limited research examining the association between social support and cognition or Alzheimer’s disease and related dementias (ADRD) blood‐based biomarkers (BBMs). We examined the longitudinal association of social support with cognition and ADRD BBMs, and whether gender or race/ethnicity modified the associations, in a large heterogenous cohort.

**Method:**

Participants included 3,601 older adults (mean [SD] age, 64.6 [8.47] years; 62.8% women, 35.2% Non‐Hispanic White [NHW], 37.1% Mexican American [MA], 27.7% African American [AA]) enrolled in the Health and Aging Brain Study‐Health Disparities (HABS‐HD). Perceived social support was measured using the Interpersonal Support and Evaluations List. Raw scores from a comprehensive battery of cognitive assessments were converted to demographically adjusted (age, education, and English/Spanish) z‐scores. These z‐scores were then used to create the following domain‐specific composites scores: memory, attention, language, and executive function. A subset of individuals (*N* =861) had BBMs (Aβ42/40, pTau181, t‐tau, NfL) measured at two timepoints, an average (range) of 2.13 (1.58 – 3.83) years apart, using the Simoa platform. Linear mixed‐effect models evaluated the longitudinal associations between baseline social support and cognitive decline or BBM trajectories. Models adjusted for baseline age, education, gender, race/ethnicity, depressive symptoms, and anxiety. Interactions between social support and gender or race/ethnicity and cognition or BBMs were assessed.

**Result:**

Higher baseline social support was associated with better performance in attention z‐scores (β=0.002, *p* =0.014), but not with other domains. Neither gender nor race/ethnicity modified the association between baseline social support and any cognitive domain z‐scores. Higher baseline social support was associated with a decrease in pTau181 levels (β=‐0.006, *p* =0.019). A significant interaction between baseline social support, gender, and pTau181 was demonstrated (β=0.013, *p* =0.010). In men, baseline social support was associated with a decrease in pTau181 levels (β=‐0.014, *p* =0.002); whereas in women no association was observed (β=‐0.001, *p* =0.755). There were no significant interactions between baseline social support, gender or race/ethnicity, and Aβ42/40, t‐tau, or NfL.

**Conclusion:**

In this large longitudinal, heterogeneous study, higher social support was associated with better cognitive performance in the attention domain and lower levels of pTau181, results differed by gender.

